# AN EXPLORATORY QUALITATIVE ASSESSMENT OF PATIENT AND CLINICIAN PERSPECTIVES ON PATIENT-REPORTED OUTCOME MEASURES AND DISEASE-MODIFYING THERAPIES IN ADULTS WITH SPINAL MUSCULAR ATROPHY

**DOI:** 10.2340/jrm.v57.41254

**Published:** 2025-01-14

**Authors:** Jeremy SLAYTER, Lauren CASEY, Shane MCCULLUM, Dorothy DROST, Allison BANKS, Colleen O’CONNELL

**Affiliations:** 1 Division of Physical Medicine & Rehabilitation, Dalhousie University, Halifax, Nova Scotia; 2 Stan Cassidy Centre for Rehabilitation, Horizon Health Network, Fredericton, New Brunswick; 3 Dalhousie Medicine New Brunswick, Dalhousie University, Saint John, New Brunswick, Canada

**Keywords:** adult spinal muscular atrophy, neuromuscular disease, patient-centred care, patient-reported outcome measures, qualitative research, activities of daily living, reliability and validity

## Abstract

**Objective:**

To understand patient, caregiver, and clinician perspectives on patient-reported outcome measures, critical functional domains, and disease-modifying therapies in adult spinal muscular atrophy.

**Design:**

An exploratory qualitative single-site study.

**Patients:**

Ten adults with spinal muscular atrophy and two clinicians participated in semi-structured interviews.

**Methods:**

Semi-structured interviews were conducted virtually or in person with participants after they completed outcome measures at a routine clinic visit. Two researchers analysed transcripts concurrently using a thematic approach to determine themes.

**Results:**

Ten themes were identified among participants. Patient-reported outcome measure preference varied between functional groups and was under-responsive, although it captured meaningful data. Motor stability was most frequently expected with disease-modifying therapy, but participants also reported improved fatigue and respiratory status.

**Conclusion:**

After considering patient goals, functional status, and preferences, patient-reported outcome measures represent a valuable adjunct to standard clinical and research tools. Optimal selection of patient-reported outcome measures requires careful consideration of multiple patient factors. Collaborative development of modified patient-reported outcome measures may yield a responsive, meaningful, and acceptable tool that can be used across a broad functional spectrum.

## LAY ABSTRACT

Clinicians and researchers need to know if they are measuring the essential items for patients. This study examines the experiences that adults with spinal muscular atrophy have with patient-reported outcome measures, which measure a person’s health status and quality of life. Through interviews with adults with spinal muscular atrophy and clinicians, we show that outcome measures need to cover broad domains of function and are also valid, responsive, reliable, and accurately capture patients’ lived experiences. Adults with spinal muscular atrophy recommended an individually tailored approach to patient-reported outcome measure selection with a renewed focus that incorporates mental health, sexual function, and fatigue, ensuring the use of the right tools at the right time. New or modified patient-reported outcome measures should be developed collaboratively with clinicians, researchers, and adults with spinal muscular atrophy to create improved and meaningful tools.

Spinal muscular atrophy (SMA) is a neuromuscular disease affecting 1 in 10,000 live births, causing lower motor neuron loss, progressive muscle atrophy, and other systemic complications (1–3). SMA is caused by homozygous deletion of the survival motor neuron 1 (*SMN1*) gene in an estimated 95% of cases (1, 3, 4). SMA has a broad spectrum of clinical phenotypes, most commonly subclassified by *SMN2* gene copy number, age of symptom onset, or functional group (3, 5, 6). Functional group classification is a variably defined but commonly accepted form of subclassification of SMA.

The therapeutic era began in 2016 with the release of disease-modifying therapies (DMT), including nusinersen (7), onasemnogene abeparvovec (8), and risdiplam (9), which altered the expected natural history and disease progression across the lifespan. Despite the accepted benefits in children, there remains relatively limited evidence supporting the use of DMT in adults, based on retrospective, observational, or real-world studies (6–13). It is also recognized that the ongoing development and refinement of outcome measures is needed, with patients, clinicians, and researchers advocating for modified outcome measures to be sensitive, reliable, and responsive to meaningful changes reported by patients to best capture treatment effects (4, 12, 14–17). As a result, the assessment and subsequent improvement of outcome measures to capture the experiences and clinical progression of adults with SMA (awSMA) has become a rapidly evolving area of research (15, 18–24).

Patient-reported outcome measures (PROMs) are emerging measures that quantify a patient’s lived experience (25–27). The Canadian Institute for Health Information defines PROMs as measurement instruments completed by patients that obtain information on aspects of the patient’s health status relevant to domains such as quality of life, symptoms, function, pain, and physical, mental, or social health (27). PROMs have been shown to have several gaps in validation for awSMA and are poorly integrated into clinical and research settings for awSMA (19, 24, 26, 28). Most currently available PROMs were repurposed to approximate the adult SMA population and may only broadly characterize a person’s level of function with emphasis on physical function, and few PROMs evaluate sexual function, speech, respiration, fatigue, and other systemic issues (17, 19, 21, 29, 30). In a PROM that is proven to be sensitive, reliable, and responsive in an adult SMA population, even mild improvement or stability over time could be meaningful compared with natural history.

Sensitive measures capable of capturing the diverse impacts of SMA are essential to providing optimal patient care and supporting informed treatment decisions (18, 31, 32). Unfortunately, traditional psychometric approaches depend on clinician or researcher expertise, with little input from patients despite established priorities to integrate patient perspectives into research (29). Understanding patient experiences and perspectives on PROMs supports the development of clinically meaningful PROMs.

This exploratory single-site qualitative thematic study aims to evaluate the meaningfulness of a selection of PROMs assessed by patients and clinicians and the feasibility of PROM implementation (33). The qualitative methodology provides a holistic and patient-centred approach that can uniquely explore patient experiences and perspectives of clinical assessments for awSMA that is complementary to traditional psychometric methods. In addition, this study sought to examine the changes patients report or expect to experience with the initiation of DMT. This study will guide future studies in exploring additional outcome measures for awSMA.

## METHODS

### Study design and participant selection

This exploratory study utilized a qualitative methodology with semi-structured interviews interpreted following a thematic approach described by Braun and Clarke (33). The authors developed the semi-structured interview questionnaire (Appendix S1), which a qualitative questionnaire specialist evaluated to minimize bias. The interview questionnaire sought to assess the perceptions of PROMs by patients and clinicians and explore patient and caregiver perspectives on their experience with SMA. During the interview, psychometric concepts, including face validity, test–retest reliability, construct validity, sensitivity, and responsiveness, were qualitatively assessed by each patient, caregiver, and clinician. The interview questionnaire also explored individual patient goals, concerns and impacts of SMA, gaps in clinical assessment, and reported effects of DMT. Interviews with clinicians explored the same concepts but integrated their clinical experiences of administering each PROM and subsequent patient interactions.

Patients were recruited consecutively from an interdisciplinary SMA clinic, which included a physiatrist, nurse, physiotherapist, occupational therapist, and respiratory therapist. Patients had to be above the age of 16 with a confirmed diagnosis of SMA and be able to complete an interview in English or French to be included in this study. A caregiver could participate during the interview if the participant preferred. Patients were excluded if they had any additional physical or cognitive comorbidities that could impact the assessment, such as concurrent neuromuscular conditions, respiratory conditions, active infections, or recent physical trauma. Once informed consent was obtained, participants would undergo the PROM assessment by a trained clinician either on the same day or upon their next follow-up visit. Participants were classified by SMA type and functional group. Walkers can walk independently with or without a gait aid for at least 4 steps, sitters can sit independently without assistance for more than 3 s, and non-sitters cannot sit unassisted for more than 3 s.

Eligible clinicians were physicians, physiotherapists, occupational therapists, or other clinical staff members of the interdisciplinary SMA clinic who had been trained in and had conducted PROM assessments as part of their clinical duties for participants in the study. Full recruitment was expected to be 15 participants. If thematic saturation was met before full recruitment, recruitment would be discontinued. Existing literature identifying common patient experiences with SMA was used to inform thematic saturation, in addition to saturation being defined *a priori* as when new participants consistently did not provide new insights, describing only what had previously been expressed (22, 34–37).

Interviews were conducted virtually via Zoom for Healthcare (Zoom Video Communications Inc) with patients and caregivers. Participants were eligible for interview after completion of all PROMs of interest. Clinician interviews were completed in person after all participants had completed the PROM assessments. Interview recordings were transcribed, de-identified, and then imported into NVivo 1.6.1 (Lumivero; https://lumivero.com/) for thematic analysis. Some direct quotes are simplified for clarity and to minimize patient health information disclosures without substantive change. Square brackets denote quote alterations by the authors.

### Patient-reported outcome measure selection and administration

PROMs were selected via a consensus meeting, which consisted of the interdisciplinary SMA clinicians (physiatrist, nurse, physiotherapist, occupational therapist, and respiratory therapist) from a group of 31 previously identified PROMs previously studied in awSMA (19). PROMs were chosen by consensus from the group by considering current clinical use, validation and reliability evidence, along with expected clinical feasibility, availability, and utility. Five PROMs were selected by consensus. The Spinal Muscular Atrophy Functional Rating Scale (SMAFRS) is a commonly recommended standard PROM (18, 38). The Spinal Muscular Atrophy Independence Scale (SMAIS-ULM) (23) and Egen Klassification Scale Version 2 (EK2) are upcoming PROMs focusing on physical function (19, 39). The Quality of Life in Neuromuscular Disease scale (QOL-NMD) (40) and the Utrecht Scale for Evaluation of Rehabilitation-Participation (USER-P) focus on quality of life in SMA and community participation (19, 41). Selected PROMs include a common standard and promising PROMs covering varying domains of function and perspectives on quality of life (19). A physiotherapist or occupational therapist administered PROMs during an interdisciplinary SMA clinic.

### Statistical analysis

Outcome measure data were collected following routine clinic data collection procedures according to previously published consensus guidelines (18). Additional demographics, including age, sex, SMA type, functional group, and treatment status, were collected during participant screening.

**Fig. 1:**
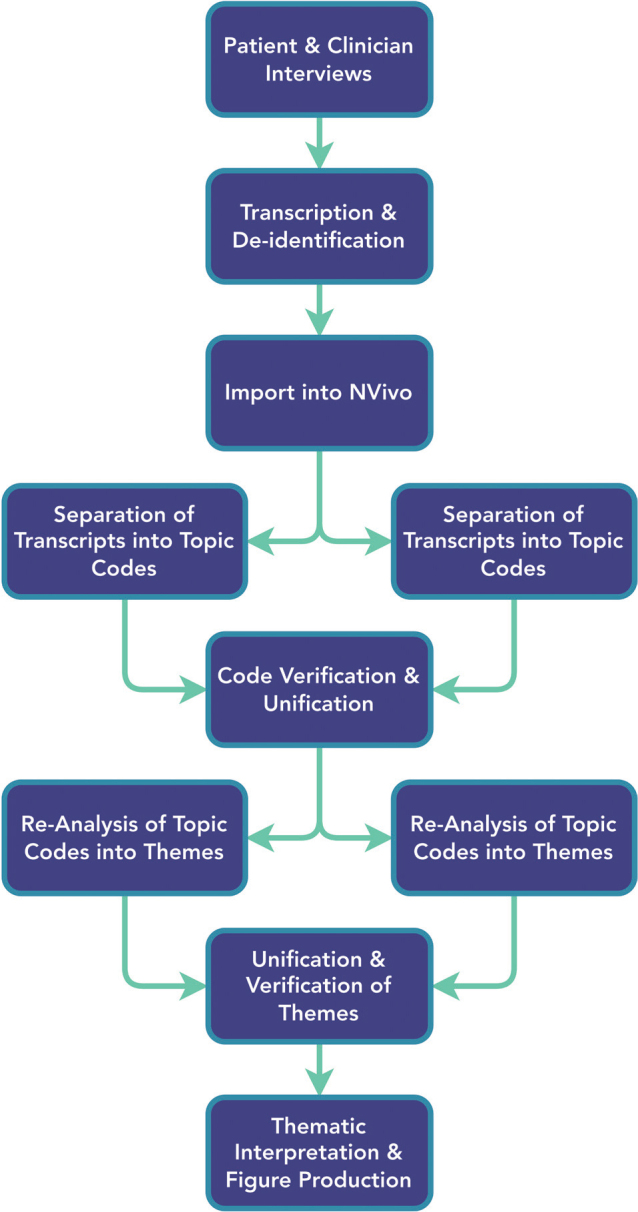
Procedural flowchart.

JS completed semi-structured interviews using the semi-structured interview questionnaire to guide the interview. Throughout the interview, participants and family members were able to review each PROM questionnaire. The interview transcripts were analysed using a thematic approach, which employed inductive and deductive reasoning during theme generation (33, 42). Thematic analysis was completed in duplicate by JS and LC and then reviewed for agreement in duplicate, as outlined in Fig. 1. Following transcript reviews, derived codes and themes were merged and re-evaluated separately by JS and LC to identify and extract additional overarching themes. In circumstances of disagreement, discussions between authors occurred to determine a final theme structure.

## RESULTS

Semi-structured interviews were conducted between March and June 2022, totalling 10 interviews with awSMA and caregivers and 2 clinicians (1 physiotherapist and 1 occupational therapist) before achieving thematic saturation. There are gaps in participant numbering as thematic saturation was met before full enrolment was achieved (Table I). Semi-structured interviews were completed a mean of 2.8 (± 2.2) months after PROM collection; 60% of participants were female, 60% were non-sitters, and 90% had SMA type 2 or 3. Most participants were receiving DMT (90%), with 5 (50%) receiving nusinersen and 4 (40%) receiving risdiplam. Of the participants receiving DMT, 4 had less than 1 year of DMT, while 5 had received DMT for between 1 and 2 years. Complete participant demographics are outlined in Tables I and II. Table III reports outcome measure scores by SMA type and functional group. Ceiling and floor effects are apparent among the EK2, SMAFRS, RULM, and HFMSE.

**Table I:** Individual participant characteristics

Participant*	Age (years)	Sex	SMA Type	Functional group	Treatment Type	SMAFRS	HFMSE	RULM
001	19	F	3	Walker	Nusinersen	48	57	37
002	55	F	3	Non-sitter	Nusinersen	14	6	22
004	40	F	3	Sitter	Nusinersen	41	35	31
005	46	M	2	Non-sitter	Risdiplam	0	0	0
006	20	M	2	Non-sitter	Risdiplam	0	NC	0
007	21	M	2	Non-sitter	Risdiplam	0	0	1
009	25	F	2	Non-sitter	Risdiplam	7	1	11
011	64	F	3	Walker	Nusinersen	45	38	37
012	19	M	4	Walker	No DMT	50	62	37
014	34	F	2	Non-sitter	Nusinersen	9	0	17
Mean	34.3 (SD 16.4)					21 (0–50)	22 (0–62)	19 (0–37)

* Participant numbers are not consecutive as patients were recruited from a concurrent study.

NC: not completed.

**Table II:** Participant demographics

	Total (n = 10)	Male (n = 4)	Female (n = 6)
Mean age in years (SD)	34.3 (16.4)	26.5 (13.0)	39.5 (17.4)
SMA Type			
Type 2	5	3	2
Type 3	4	0	4
Type 4	1	1	0
Functional population			
Non-sitter	6	3	3
Sitter	1	0	1
Walker	3	1	2
Disease modifying treatment			
Nusinersen	5	0	5
Risdiplam	4	3	1
No treatment	1	1	0

**Table III:** Outcome measure score by SMA type and functional group

Item	EK2 (*n* = 9)	QOL-NMD (*n* = 8)	SMAFRS (*n* = 10)	SMAIS-ULM (*n* = 10)	USER-PF (*n* = 9)	USER-PR (*n* = 9)	USER-PS (*n* = 9)	RULM (*n* = 10)	HFMSE (*n* = 9)
Min–Max Score	0–51	0–75	0–50	0–44	0–100	0–100	0–100	0–37	0–66
Type 2	30.6 (11.4)	45.5 (14.66)	3.2 (4.44)	10.2 (10.71)	28.56 (9.44)	42.54 (10.32)	75.94 (15.74)	5.8 (7.79)	0.25 (0.5)
Type 3	4.7 (4.73)	47.7 (0.58)	38.3 (13.1)	40.5 (4.51)	31.2 (13.50)	58.2 (3.18)	67.7 (18.01)	31.8 (7.09)	34 (21.06)
Type 4	0	75	50	44	55	97	100	37	62
Non-sitter	27.2 (13.24)	46 (12.75)	5.8 (7.57)	14.2 (13.64)	26.7 (9.65)	44.5 (10.44)	71.6 (17.62)	8.5 (9.61)	1.4 (2.61)
Sitter	1	48	41	44	44	60	67	31	35
Walker	1.5 (2.12)	61 (19.80)	47.7 (2.52)	42.7 (1.53)	43.75 (15.91)	78.5 (26.16)	93 (9.90)	37 (0)	52.3 (12.66)
Overall	18.56 (16.64)	50 (13.98)	21.9 (21.62)	25.7 (18.05)	32.4 (12.80)	53.8 (19.38)	75.9 (17.41)	19.3 (15.77)	22.1 (25.98)

Values reported are mean (SD).

EK2: Egen Klassification 2 Scale; QOL-NMD: Quality of Life in Neuromuscular Disease; SMAFRS: Spinal Muscular Atrophy Functional Rating Scale; SMAIS–ULM: Spinal Muscular Atrophy Independence Scale–Upper Limb Module; USER–PF: Utrecht Scale for Evaluation of Rehabilitation–Participation Frequency Subscale; USER–PR: Utrecht Scale for Evaluation of Rehabilitation–Participation Restriction Subscale; USER–PS: Utrecht Scale for Evaluation of Rehabilitation–Participation Satisfaction Subscale; RULM: Revised Upper Limb Module Subscale; HFMSE: Hammersmith Functional Motor Scale – Expanded.

**Fig. 2:**
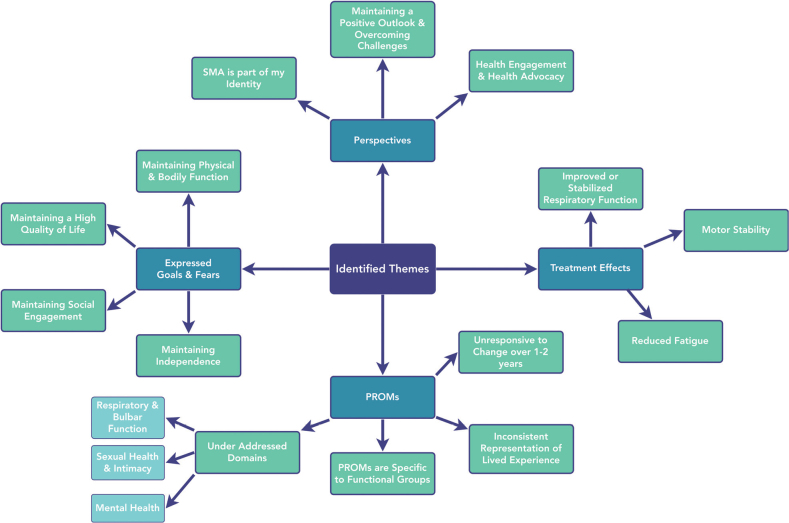
Thematic concept map.

Four thematic groups were identified: themes surrounding individual goals and fears, perspectives on disease, perspectives of PROMs, and perceived treatment effects of DMT (Fig. 2). Across the 4 thematic groups, 10 themes were identified from an initial 1,191 code references and 356 thematic code references.

### What are the concerns and impacts of SMA?

*Theme 1:* Maintaining bodily function, minimizing limitations, and maximizing social and workplace participation are significant for adults with SMA.

*Theme 2:* Adults with SMA maintain a positive perspective, searching for solutions to overcome barriers.

Participants described many physical and social concerns arising from SMA. The most frequent concerns were losing motor, respiratory, or bulbar function, which could threaten their independence or identity. Physical concerns were losing the ability to walk or climb stairs, complete activities of daily living (ADLs), maintain fine motor function, drive a vehicle, breathe, or swallow. Social concerns were the inability to participate in their community, navigate the education system, and obtain a post-secondary education, loss of intimacy, health anxiety, and difficulty communicating. Despite these concerns, participants report high overall life satisfaction and felt equipped to overcome diverse challenges through statements such as “it’s my normal, I don’t know anything different” (001), and “I’m a person that always tries to find solutions” (011).

### Are PROMs acceptable, meaningful, and responsive to change?

*Theme 3:* Currently available PROMs are qualitatively described as under-responsive to patient-identified meaningful functional changes.

*Theme 4:* PROMs explore meaningful topics but must address patient functional priorities.

*Theme 5:* PROM acceptability is specific to functional groups; walkers tended to prefer broad PROMs, while non-sitters preferred disease-specific and task-specific PROMs.

*Theme 6:* PROM development and modification should incorporate a patient-centred approach to design and implementation.

Participants reported that PROMs explored meaningful topics, although they were under-responsive to meaningful changes or needed to be more consistent in their comprehensiveness, particularly when stratified across functional populations and ages. Amongst walkers, the SMAFRS and QOL-NMD were the most meaningful and best assessed daily function. At the same time, non-sitters reported that the SMAFRS poorly represented their abilities. Participant (005) stated, “It does a good job at capturing what I can’t do”, and a caregiver for 006 stated, “As a parent, sitting there and people asking, ‘if they can do this, can they do that?’ … We know what they can do and what they can’t. I found it to be really depressing, and I couldn’t wait to get out of there.” Participants recommended that PROMs incorporate how a task was completed or utilize a relative reference frame when asking questions, such as comparing their function through time. Participant 011 highlighted the under-responsiveness of PROMs and the utility of a relative reference frame when they stated, “I know I can’t do things I could do last year … like my cottage. I only went there in the summer. So, a whole year goes by, and I knew that I could do that last summer, that wasn’t hard or not that bad. It was manageable. Now, I can’t do those stairs without help. So, I knew, it’s just a tiny bit of a change and yes it might be just a tiny bit on your measurements, but to me, no.” Similarly, 5 other participants described similar experiences across 15 statements.

**Fig. 3:**
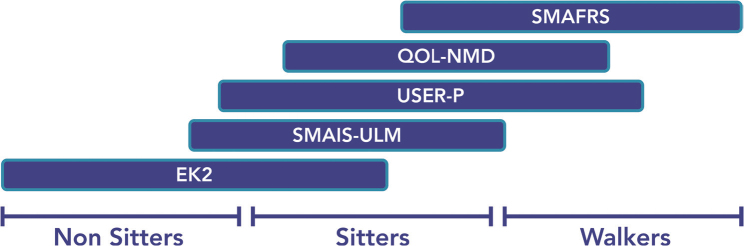
Proposed PROMs based on functional spectrum.

PROM acceptability varied by functional group, where a personalized approach to PROMs, which accounted for functional status, was felt by participants to better approximate a person’s experience. Participant 001 described the need for a personalized approach to PROMs by stating, “The questions are very much for SMA type 2 or 3. I don’t really have as big of a challenge as what the questionnaires were asking. You won’t see a lot of change for me in those questionnaires”. In contrast, participant 006 reported, “To constantly be run through those questions and hearing zero, zero, zero, zero, zero… I just found it to be really hard.” PROMs were most acceptable and responsive for participants with a more rapid disease course who currently have moderate levels of function, as opposed to participants on either end of a functional spectrum (Fig. 3).

Upon examination of individual PROMs by participants and clinicians, each has apparent strengths and weaknesses. The QOL-NMD best addressed psychological status and had relatively broad domain coverage, although it inadequately addressed respiratory status and had poor responsiveness. The EK2 and SMAIS-ULM were the most responsive measures and were adequately detailed within assessed domains, although neither was comprehensive in domain coverage. The EK2 was most accepted in the non-sitter and sitter populations due to a focus on fine motor function. However, there were concerns about it being discouraging to complete; Participant 005, a non-sitter, stated that the EK2 is the “worst measure” because of “the scoring of zero to four. I have my day, and I get my results, and it’s basically a zero, so it’s kind of the opposite of motivational.” Participant 004 also noted that, “for certain people, it may bring up some negative stuff”. Clinicians also reported that scoring the EK2 could be challenging to interpret due to the reversed scoring system, although it was easy to complete and was an appropriate length.

Sitters and non-sitters accepted the SMAIS-ULM, which was easy to complete and an appropriate length. Clinicians reported that the SMAIS-ULM was easily scored and provided a detailed assessment of upper limb function, although it did not cover other functional domains. Sitters and non-sitters accepted the USER-P, which accurately captured most ADLs, although at times was unclear and lacked responsiveness. The SMAFRS was frequently described as discouraging, owing to poor responsiveness and apparent ceiling and floor effects when assessing participants at either end of the functional spectrum (7 codes). Overall, participants felt that the SMAFRS did not provide meaningful results, although it was comprehensive in domain coverage (C002, P006). As reflected in Table III, clinicians reported a prevalent floor effect with the SMAFRS in non-sitters. Clinicians additionally found the SMAFRS to be poorly responsive, although acknowledged that it may be able to best stratify functional groups compared with the remaining PROMs (C001, C002).

Clinicians reported the SMAIS-ULM to be the “most sensitive for [the patients with] middle-of-the-road abilities” (C002). Despite the SMAIS-ULM being relatively responsive to evaluated PROMs, clinicians believed that the SMAIS-ULM may only detect changes seen after a 5-to-10-year interval, varying by SMA type and disease progression. Clinicians described the EK2 as the most clinically meaningful due to its broad and detailed coverage of a person’s physical abilities. Clinicians highlighted the risks of introducing unintentional confounders in the QOL-NMD and USER-P questionnaires as a person’s overall function is not solely a function of their disease but is the product of a complex interaction between the medical, community, financial, and social supports available.

### What are the current gaps in PROMs assessment?

*Theme 7:* PROMs should incorporate additional domains of function, including mental health, intimacy, sleep, and bulbar function.

*Theme 8:* PROMs may not wholly capture an individual’s life satisfaction and functional abilities.

PROMs covered the most meaningful domains of function, although participants and clinicians highlighted gaps. Mental health, intimacy, fatigue, sleep, and bulbar function were recognized as essential gaps that tend to be inadequately addressed amongst the evaluated PROMs. Participant 004, in a discussion on mental health, stated, “Trying to deal with everything we have to deal with, it takes a toll on your mental health”, and then later reported that they had “always had to figure that [mental health] out on [their] own”. Clinicians highlighted “tunnel vision” occurring, where clinicians chose tools to capture what they felt was essential but can miss other functional domains such as mental health. Clinicians reported that global cognitive ability was also poorly captured by the evaluated PROMs.

Clinicians also described scenarios of patients who had scored low on physical domains. However, patients continued reporting high levels of function with assistance from formal and informal supports, such as maintaining an occupation and achieving their life goals. This highlights a potential gap between individuals’ measured and actualized overall function.

### What goals and effects do participants with SMA report from DMT?

*Theme 9:* Adults with SMA hope to achieve overall disease stability with treatment.

*Theme 10:* Adults with SMA receiving DMT reported improvement or stability in motor function, respiratory status, bulbar function, and fatigue levels, allowing them to perform tasks easier and for longer than previously.

The most frequently described goal by awSMA was to achieve stability or improvement in gross and fine motor function. Maintaining the independence to partake in different activities and complete ADLs was a unifying theme, with commonly described goals including maintaining upper limb strength and dexterity, respiratory function, and independence. In discussion around goals, Participant 005 stated, “It’s being a member of society, being able to work, to socialize, to get out of bed and enjoy the day, as opposed to the individual physical tasks”. Ambulatory participants sought to maintain the ability to walk and be active with reduced fatigue.

Participants endorsed numerous changes in their function since starting DMT, including improved respiratory and bulbar function, disease stability, strength, coordination, and fatigue. Participant 004 described stability and functional improvements by stating, “I was able to do [physical tasks] before, but now I am doing them better or easier”. Regarding respiratory and bulbar function, participant 006 and their caregiver stated, “But ability to cough? That might be something that could change because I question whether the drug has helped their respiratory status because 006 has [had frequent respiratory illnesses]. And … this year, they have not. Their first time ever.” With these noted changes, some participants reported having fulfilled their treatment expectations. Participants 001 and 004 described achieving stability in motor function for up to 36 months since DMT initiation, while 3 participants noted improved respiratory health. Others noted improved phonation and swallowing (Participants 005, 007).

## DISCUSSION

The DMT era has permanently shifted the therapeutic landscape for awSMA. Adapting to the modern environment requires clinicians and researchers to consider additional outcome measures representative of SMA’s changing patient experience and expected disease progression. PROMs represent an emerging solution to capture patient-centred data on functional domains that have been poorly captured. Despite their potential, the assessed PROMs focused heavily on physical function while infrequently addressing other functional domains that holistically impact a person’s life.

Motor function has been well established as one of the primary concerns of awSMA, as motor function supports the ability to work, interact with the environment, complete ADLs, and maintain one’s independence (22, 29, 34). The literature supports the results of this study, as gross motor function was the most described concern amongst walkers (34). In contrast, fine motor function became most important in the sitter and non-sitter groups. This finding is in keeping with previous literature, as motor dysfunction is a hallmark clinical finding of SMA via lower motor neuron loss (22, 29, 34).

Mental health and fatigue were under-captured by the evaluated PROMs, with only the QOL-NMD explicitly assessing components of mental health. Further, participants reported that they have traditionally managed alone, despite increasing recognition by clinicians that the presence of chronic conditions increases the risk for poor mental health, which is known to affect function in a myriad of ways (43, 44). Further research should be undertaken to identify optimal PROMs that adequately assess mental health, as it is an important domain for awSMA (19). Participants additionally highlighted that fatigue can be a daily challenge, often seeking to lessen fatigue to complete ADLs efficiently.

Clinicians and participants described scenarios of individuals scoring low on PROMs due to the severe physical nature of the disease. However, they remained active members of society, maintained an occupation, engaged in their community, completed leisure activities, and led fulfilling lives. Participants additionally highlighted the need for improved assessment of their overall function, as they felt discouraged following an evaluation if they had consistently scored low, despite feeling satisfied with their abilities. The dichotomy of lived experience against assessment scores highlights a limitation that PROMs may inadequately represent an individual’s functional status; therefore, important functional domains to capture patient experiences may continue to be under-recognized with current PROMs.

PROM acceptability and perceived utility depended on the functional group. The clinician should select PROMs based on the functional group to optimize PROM utility and limit the risk of floor or ceiling effects. With poor PROM selection, participants can become discouraged (often secondary to floor or ceiling effects), feel that they may face future substantial functional decline, or think that the measure is not representative of their abilities. Among sitters and non-sitters, preferred PROMs were disease-specific, emphasizing fine motor function and ADLs, with them often preferring the SMAIS-ULM and EK2 (Fig. 3). Walkers preferred the SMAFRS, USER-P, and QOL-NMD, primarily assessing gross motor function or broader functional status. For patients at the extremes of the functional spectrum, PROMs unintentionally could be harmful by failing to capture the patient’s perspectives accurately.

PROMs may be most sensitive to change for patients in the middle of the functional spectrum; however, patient perspectives may need to be captured more adequately. PROM accuracy can be affected by how questions are asked, as adaptations, altered supports, or task modifications can add complexity to responding to questions, changing the clinician’s interpretation of PROMs. PROMs can fail to address these subtle nuances – although, with careful measure design, this can be mitigated by integrating techniques such as ratings of perceived exertion or by employing a relative reference frame during responses. Alternative options to reduce ambiguity may include adding an open-ended response or including concurrent completion of functional outcome measures. PROMs remain a viable assessment tool based on patient and clinician experiences outlined in this pilot study. However, they require substantial refinement to achieve accurate and representative functional monitoring for awSMA.

### Limitations

The qualitative methodology of this study does not provide quantifiable psychometric data of the studied PROMs for validation, reliability, or responsiveness; however, this study presents a unique patient-centred approach to understanding patient perspectives that cannot be obtained through traditional psychometric methods. The small sample size and heterogeneous group of patients across a broad spectrum of functional abilities could limit identified themes for specific population subsets. However, thematic saturation was achieved before full study recruitment, suggesting an adequate sample size despite the small number. Further, given this study’s qualitative nature, a large quantity of rich interview data supports further hypothesis generation. The results of this study encourage and inform future research into the identified weaknesses of PROMs and potential areas for additional development. Another limitation of this study was the gap between the completion of PROMs and interviews, which could impact the memory of completing each PROM. Participants could freely review each PROM during the interview to reduce the risk of poor recall. Despite these limitations, the authors believe this study provides a unique perspective on an emerging topic for awSMA.

### Conclusions

This study provides a unique perspective on patient-centred data examining available PROMs for awSMA. PROMs represent an emerging option to monitor functional status in SMA, providing valuable information that may otherwise be missed. PROMs should be carefully selected, using a shared decision-making process to best support patients’ goals, expectations, and values. Inappropriate selection of PROMs can lead to increased risks of psychological harm, reduced health engagement, and overall patient discouragement.

Our findings that PROMs underperform patient expectations are consistent with the literature (24). We suggest that PROMs should be considered adjunct measures to supplement the clinical history, physical examination, and other outcome measures, recognizing that established functional measures or biochemical biomarkers may provide a more reflective, responsive, and valid approach. Further exploration of composite measures, including PROMs, motor measures, and biomarkers, may expand outcome measure utility to better capture a broad spectrum of function. To further improve PROM performance, current measures should be modified, or new measures should be developed to target a pre-defined population, with goals established in a collaborative setting between clinicians, researchers, and patients to create a measure that allows in-depth and meaningful evaluation of a person’s lived experiences.

## Supplementary Material

AN EXPLORATORY QUALITATIVE ASSESSMENT OF PATIENT AND CLINICIAN PERSPECTIVES ON PATIENT-REPORTED OUTCOME MEASURES AND DISEASE-MODIFYING THERAPIES IN ADULTS WITH SPINAL MUSCULAR ATROPHY
