# Supplementary material for: AN EXPLORATORY QUALITATIVE ASSESSMENT OF PATIENT AND CLINICIAN PERSPECTIVES ON PATIENT-REPORTED OUTCOME MEASURES AND DISEASE-MODIFYING THERAPIES IN ADULTS WITH SPINAL MUSCULAR ATROPHY
Source: J Rehabil Med. 2025 Jan 14;57:41254. doi: 10.2340/jrm.v57.41254 (PMC11748171; doi:10.2340/jrm.v57.41254)
Supplement: AN EXPLORATORY QUALITATIVE ASSESSMENT OF PATIENT AND CLINICIAN PERSPECTIVES ON PATIENT-REPORTED OUTCOME MEASURES AND DISEASE-MODIFYING THERAPIES IN ADULTS WITH SPINAL MUSCULAR ATROPHY [file JRM-57-41254-s1.pdf]

Supplementary material has been published as submitted. It has not been copyedited, or typeset by Journal of Rehabilitation Medicine

Appendix S1
PROM Interview Questions

Patient ± Caregiver Interview Questions

1. Tell me about your experience with your diagnosis of Spinal Muscular Atrophy.
    a. What has living with SMA been like?

2. What are some important parts of your daily life and routine?
    a. Probe: Tell me what a typical day is like for you.
    b. Probe: What do you like to do?

3. Could you tell me about what you fear you might lose due to your disease?
    a. Probe: This could be the physical ability to do something or anything that you have thought about; there is no wrong answer.

4. **IF ON Treatment:** What is a meaningful change that would make you think that treatment was helpful?

5. Moving now to talk about the assessments which were recently completed; do you feel as though these assessments are useful?
    a. Probe: How do you think they capture your current capabilities?

6. What kind of assessments do you think would be helpful in tracking your experiences with SMA?

Repeat questions 7 to 13 for each PROM

7. From your perspective, what does this assessment do well? Why?

8. From your perspective, what could this assessment do better?

9. From your perspective, what are things which are important to you but are not covered?

10. Do you think that this assessment captures meaningful aspects of your life?

11. Was the assessment challenging in any way? challenging for you to do, tiring or difficult to understand?

12. How did you find the amount of time it took to do the assessment?
    a. Probe: Was the assessment too long or too short?

13. Do you feel as though the assessment would be able to capture changes in your abilities?

14. Is there anything else you would like to tell me about your experiences with SMA or assessments?

15. Are there any questions we should ask people that weren't included here?

Clinician Interview Questions

1. From your perspective, what are things which are important for patients and are routinely covered by the assessments?

2. From your perspective, what are things which are important for patients but are not routinely covered by the assessments?

Repeat questions 3 to 7 for each PROM

3. From your perspective, what does this assessment do well? Why?

4. Do you think that these assessments are clinically meaningful?

5. From your perspective, what could this assessment do better?

6. Was the assessment challenging for you to do, tiring for the patient or difficult to understand? Was the assessment too long or too short?

7. Do you feel as though the assessments used are able to capture changes in patient abilities?

8. Is there anything else you would like to tell me about on these topics?

9. Are there any questions that we should be asking people that weren't included here?
